# Research on the Effects of Gender and Feeding Geese Oats and Hybrid Rye on Their Slaughter Traits and Meat Quality

**DOI:** 10.3390/ani11030672

**Published:** 2021-03-03

**Authors:** Dariusz Lisiak, Piotr Janiszewski, Eugenia Grześkowiak, Karol Borzuta, Beata Lisiak, Łukasz Samardakiewicz, Tomasz Schwarz, Krzysztof Powałowski, Krzysztof Andres

**Affiliations:** 1 Department of Meat and Fat Technology, Prof. Wacław Dąbrowski Institute of Agricultural and Food Biotechnology, 60-111 Poznań, Poland; dariusz.lisiak@ibprs.pl (D.L.); eugenia.grzeskowiak@ibprs.pl (E.G.); karol.borzuta@ibprs.pl (K.B.); beata.lisiak@ibprs.pl (B.L.); lukasz.samardakiewicz@ibprs.pl (Ł.S.); krzysztof.powalowski@ibprs.pl (K.P.); 2 Faculty of Animal Science, University of Agriculture in Kraków, 31-120 Kraków, Poland; tomasz.schwarz@urk.edu.pl (T.S.); krzysztof.andres@urk.edu.pl (K.A.)

**Keywords:** geese, feeding, rye, oats, slaughter value, quality traits

## Abstract

**Simple Summary:**

The research showed the influence of the gender of geese on some slaughter value traits and meat quality of geese slaughtered at the age of 17 weeks. It was found that ganders had a greater body and carcass weight than females but no significant differences in slaughter yield nor in carcass element share were observed. Gender did not have an effect on the majority of the studied breast meat quality traits. The studied feeding model did not have a significant influence on goslings’ body weight; however, the birds fed hybrid rye had a lower slaughter yield as compared with those fed oats, but their meat had better physical and chemical characteristics (lower fat content, lower drip loss, higher protein content and, in the female goslings, also better sensory quality). Hybrid rye may be used in geese feed because it does not have a negative effect on pre-slaughter body weight and has a positive effect on some meat quality traits as compared with feeding geese oats.

**Abstract:**

The aim of the study was to determine the effect of feeding Zatorska variety geese hybrid rye, oats, or a mixture of both grains (1:1) on slaughter value and meat quality. At 14 weeks old, the birds were separated into three feeding groups (*n* = 12) and were fed between 15 and 17 weeks of age with hybrid rye, oats, or a mixture of these two grains. The research proved the effect of gender and feeding on some slaughter value traits and meat quality of the goslings’ breast meat. It was found that the ganders had a 10% to 15% higher body and carcass weight than the females. No significant differences were observed between the genders within the majority of the physical and chemical characteristics as well as the sensory traits. The feeding type did not have a significant effect on the goslings’ body weight and carcass element share. The birds fed hybrid rye had a 2 percentage points lower slaughter yield than those birds fed oats which was combined with a lower share of subcutaneous fat (measured as the weight of the tissue coming from dissection) in birds fed hybrid rye. The meat of the birds fed hybrid rye had some better physical, chemical characteristics and, in the female goslings, also better sensory quality. The results indicated that hybrid rye may be used in feeding goslings at the end of the growing period, because it did not cause any negative effects on the pre-slaughter body weight and had a positive effect on some meat quality traits, such as better sensory estimation results, higher protein content, and lower drip losses.

## 1. Introduction

The share of geese in the poultry production sector in Poland is at a low level, amounting to just 1.5%. Approximately 95% of the geese population in mass production are the White Kołudzka geese bred in the Experimental Center of the National Research Institute of Animal Production in Kołuda Wielka [1,2,3,4], originating from the white Italian geese. Purebred animals may still be found on some breeding farms especially in the flocks under the protection program.

The results of many studies have proven that genotype has a significant influence on tissue content of carcass and chemical content of meat, as well as the muscle structure [3,5]. For instance, geese from rare breed flocks have a lower body weight, better musculature, and a lower fat content as compared with the popular white Kołudzka geese [2,3,5,6].

The Zatorska variety geese, kept in the Experimental Center of the Agricultural Academy in Cracow, belong to the Southern varieties which are used in the protection program.

In a study by Kapkowska et al. [7], the results of fattening the Zatorska and white Kołudzka geese by feeding oats between 14 and 17 weeks were compared, proving that the Zatorska geese body weight (both sexes) in the 17th week of age (5648 g) was significantly lower than the white Kołudzka geese body weight (6814 g). Moreover, the Zatorska geese had a lower slaughter yield and higher thigh and shank muscle share. Gomułka et al. [8] estimated the microstructure of the pectoralis superficalis and biceps femoris muscles and determined the technological parameters of the birds’ meat at the age of 17 weeks after fattening by feeding oats. The muscle microstructure seemed to be similar in the Zatorska and white Kołudzka geese apart from the higher content of type I muscle fibres in the M. pectoralis superficialis in the Zatorska geese. Similar values for the majority of the physical traits for the muscles of both groups of geese were observed [8].

Geese fed oats have been the subject of many studies and publications [9,10,11,12]. Rye is traditionally regarded as a grain of limited suitability in poultry feeding especially in young slaughter birds. The reason for these limitations is the negative impact on the digestive system because of higher food content viscosity, the lower speed rate of the movement of intestinal digestion, and the lower digestibility of nutrients. Such observations may lead to negative effects in terms of poultry production and welfare factors [11]. In recent years hybrid varieties of rye have appeared on the market such as Brasetto which has better agrotechnical features. Furthermore, hybrid rye has a lower anti-nutritional substance content especially with low starch polysaccharides (NPS). NPS present in grains are among others pentosans (xylans and arabinians) as well as beta glucan. Rye grain is especially rich in arabinoxylans (AX) that are not digested by monogastric animals due to the lack of suitable enzymes [13,14]. The AX structure varies because of many factors, i.e., the grain type and variety. The various AX structures have an impact on the functional features and anti-nutritional effect level [13,14].

The research of Świątkiewicz and Arczewska-Włosek [14] showed that the Brasetto hybrid rye grain can be used in the feeding of young chickens, since it is a useful energy and protein source. Introducing 15–20% rye into the feeding mixture for older birds (from 22 days of age) contributed to obtaining the desired production results, while introducing Brasetto rye added to the feed mixture for smallest chickens (1–21 days) had a negative effect on daily gain [13].

The aim of this study was to determine the effect of gender and feed mixture on slaughter value and physical, chemical, and sensory traits of the meat of birds fed hybrid rye, oats, or a mixture of these two grains.

## 2. Material and Methods

### 2.1. Experimental Material

The experiment was performed according to the guidelines issued by the Ethics Commission (Regulation 22/2016 of 20 January 2016r ILKE in Cracow). The experiment was performed on the Zatorska variety geese. This is a Polish rare breed meat type that is under the genetic resource protection program.

The geese were hatched from hatching eggs (Brinsea Ova-Easy 380) that belonged to the Experimental and Education Centre of WHBiZ UR (Faculty of the Animal Science University of Agriculture, Cracow, Poland). The experimental flock was made up of 300 young geese that were reared according to the slaughter geese rearing regulations in Poland. Geese reared in Poland in the last 3 weeks of fattening are fed only oats and water. Oats, due the specific chemical composition, characterized by a high fat content and the profile of fatty acids profitable for human health, increase the value of goose meat and fat [2].

After hatching on 18 July 2017 and after gendering, the young geese were moved to a nursery where they were kept on straw bedding with natural light and with additional artificial lighting for 24 h in the first week of life.

For the first 3 weeks, the geese did not have access to pasture and the density was 7 geese per m^2^. The temperature in the nursery was lowered from 28 °C to 22 °C within this period and the relative humidity ranged between 65–70%.

### 2.2. Feeding

The plan of the experiment is presented in Table 1 below.

For the first 3 weeks, the geese had access to feed and water through the adjusted to their age troughs and semi-automatic drinking bowls. The geese were fed dry complete feed of the proper content and nutrients which are presented in Table 2.

Between the 4th and 14th week of age, the geese were kept on straw bedding with 3.6 goslings/m^2^ stocking density in a building with windows. The geese had access to pasture, with 12.5 m^2^ space for each bird, and they stayed on the pasture for at least 8 h a day. The temperature in the nursery building was 20–22 °C and the relative humidity ranged between 65–70%. Between the 4th and 14th week of age, the geese were fed a mixture similar to the feed provided to geese between 0–3 weeks of life but additionally enriched with sunflower meal and wheat bran (Table 2).

After reaching 14 weeks, the birds were weighed and divided into groups according to gender and randomly divided into 3 feeding groups for each sex (*n* = 12). The birds of each group were fed ad libitum with grains according to the following schemes:

**Group A**: Brasetto hybrid rye with the chemical composition according to Świątkiewicz and Arczewska Włosek [13];
**Group B**: Oats, according to the composition and nutritional recommendations for geese [14,15];
**Group C**: A mixture of the Brasetto hybrid rye and oats (1:1 by weight).

The fattening was performed in pens of 1 bird/m^2^ density on the floor system, in a building with windows, on straw bedding, with 8 h access to straw pasture (stocking density 1.2 m^2^/bird). The temperature in the building was 12–18 °C. and the relative humidity ranged between 65–70%.

In the 17th week of life after being fattened by feeding grain, the birds were marked and not fed for 10 h because emptying the intestines has an impact on the quality and shelf life of carcasses during storage. After no food for 10 h, each bird was weighed individually, and then slaughtered in the commercial slaughter plant. The carcasses, after gutting, were chilled using the blowing and spraying method. Then, each carcass was weighed individually. The slaughter yield was calculated according to the following formula: MT/MC × 100%, where MT is a weight of chilled carcass and MC body weight before being slaughtered.

### 2.3. Dissection

The cut-up of the carcasses was performed according to the Ziołecki and Doruchowski [16] method for determining yield of the following parts: breast muscles, leg muscles, skin with fat, abdominal fat (from the bottom part of the abdomen), neck with fat (cut between the last cervical vertebrae and the first thoracic vertebrae), and wings (cut at the shoulder joint). Each carcass part was weighed and its share in the total chilled carcass was calculated.

### 2.4. Physical and Chemical Characteristics of Breast Muscles

Twenty-four hours after slaughter, the pH was measured using a Mettler Toledo 1140 type pH meter with a Mettler Toledo electrode (Mettler Toledo, OH 43240, D.C., US). The meat colour was determined on the muscle cross-section using a Konica Minolta Chroma CR 400 tool (Konica Minolta, Tokyo, Japan). Colour was classified according to the CiE lab determining L* (lightness), a* (redness), and b* (yellowness), with the following measurement parameters: light source D65; observer 2°; measuring head slot 8 mm; and calibration on the white tile L* = 97.83, a* = 0.45, and b* = 1.88.

The samples of the final products were taken for lab analysis. The water content was measured according to the ISO 1442 (2000) [17]. Approximately 3 g of the minced meat was put on a weighing dish, weighed, and dried at a temperature of 105 °C up to the moment when the stable mass was reached. The water content expressed in % was calculated as a difference between the sample weight before and after drying.

The intramuscular fat content was established according to the ISO 1444 (2000) procedure [18]. The dried and weighed sample was placed in an extraction tube and the fat substances were extracted with paraffin oil in a Soxtherm device produced by the Gerhardt Laboratory System (Gerhard GmbH & Co, Konigswinter, Germany). The fat content was calculated as the difference between the sample weight before and after extraction. The protein content was established according to the Polish norm PN/A-04018 [19] with a Kjeltec System 1002 Distilling Unit (Foss, Hilleroed, Denmark), according to the manufacturer’s instructions. The sodium chloride in the final products was established according to the ISO 1841-2 (2002) procedure.

Apart from measuring the colour with a device it was also determined visually using a pattern with a 1 to 5 scale (1, light pink colour to 5, dark red colour) [20].

Similarly, meat marbling was estimated on the muscle cross-section according to a pattern with a 1 to 5 scale (1, slight fat content to 5, high fat content [20].

Drip loss was determined as follows: A muscle sample of approximately 100 g was weighed, placed in a plastic bag, and left in a refrigerator at a temperature of 4 °C for 48 h. The drip loss was calculated and based on the weight difference before and after storage.

Cooking loss was determined as follows: A muscle sample of approximately 150 g was weighed. Next, it was heated in water up to a temperature of 75 °C in the geometric sample centre. After cooling, the sample was weighed, and the weight loss was calculated based on the difference between the weights before and after cooking [21].

### 2.5. Sensory Test

A sensory test of cooked meat was performed by a team of 5 people trained in terms of sensory sensitivity with the Baryłko-Pikielna and Matuszewska method [21]. The estimation was performed in daylight at room temperature. Meat flavour, juiciness, tenderness, and palatability were determined using a 5-point scale [22]. In the cooked meat samples, meat tenderness was measured by using the Warner–Bratzler shear force method in a Zwick Roell Z 0.5 device (Zwick Roell, Ulm, Germany) with 500 (Kilonewton—kN) force and 100 mm/min of head movement.

### 2.6. Statistical Analyse

The results were statistically developed by calculating mean values (x) and standard error of the arithmetic mean (SEM). The statistical significance of the differences between the mean values of groups was verified by using a two factors variance analysis according to Anova procedure using the Statistica program Version 13 (StatSoft Hamburg, Germany).

## 3. Results

The geese body weight before slaughter in all of the feeding groups was at a similar level (Table 3). Clear differences were found between the genders (*p* *≤* 0.01). The ganders reached a higher body weight than the young female geese (by approximately 10–15%). A similar effect was observed for carcass weight, but the effects of feeding was confirmed here in the group of young female geese that were heavier in the group fed oats than in the group fed hybrid rye (*p* *≤* 0.05). A significantly lower slaughter yield was observed in both genders fed hybrid rye than those fed oats and the difference between the groups was over 2 percentage points (pp). The slaughter yield of those geese fed a mixture of both grains did not differ significantly as compared with the birds that were fed hybrid rye or oats only.

The weight and the percentage share of the basic carcass parts are presented below in Table 4. The weight of these parts was significantly different between the sexes (*p* ≤ 0.01), except for body frame weight in the oats group. The higher weight of the other parts was observed in the ganders due to their higher carcass weight. The feeding method had a significant impact on the leg weights of the young female geese which were heavier in the group fed oats than those fed hybrid rye and a mixture of both grains (*p* ≤ 0.01). The carcass parts yield did not depend on the gender and the feeding method, except for the higher share of wings in the ganders fed hybrid rye.

The average weight and share of the chosen muscles and fat is presented below in Table 5. No significant differences between the dissection elements were found in terms of sex and feeding method, except for leg muscle weight and skin with fat weight. The muscle weight from the ganders’ legs was approximately 17% higher than in the young female geese (*p* ≤ 0.01). The weight of the meat from the legs was significantly higher in the female geese fed oats (*p* ≤ 0.05) than those fed hybrid rye or a grain mixture. The weight of skin with the fat of females was lower in those birds fed hybrid rye only, but the share of this element was the same in all groups. A no gender not feeding method effect was confirmed on the weight and percentage share of breast muscles and abdominal fat. The only exception was the group of birds fed a mixture of both grains in which the breast muscle weight of the ganders occurred to be higher than in the female geese (*p* ≤ 0.05) and the abdominal fat share was higher than in the female geese (2.04% and 2.94%, respectively).

The results of the breast muscle physical parameters measurements are presented below in Table 6. Lower average pH_24_ values were reported only in the muscles of geese fed oats (5.88). No significant effect of sex and feeding method on colour lightness L* and colour parameters (a* and b*) was observed. These parameter values were similar with the following ranges: L* from 35.60 to 36.52, a* from 18.39 to 20.15, and b* from −1.76 to −0.95. Generally, these measurements were confirmed by a visual colour estimation of the muscles of the birds, both genders, fed hybrid rye or oats (3.12 to 3.38 points). However, the muscles of the female geese fed a mixture of both grains were lighter (2.97 points) than those fed hybrid rye (3.28 points).

The meat marbling of both sexes was significantly influenced by the feeding method (*p* ≤ 0.01). Greater marbling was observed in the muscles of birds in both sexes fed oats (1.6 points) than those fed hybrid rye (1.32 points). In addition, birds fed a mixture of both grains had greater marbling than those fed hybrid rye (*p* ≤ 0.05) but only in the case of female geese (1.53 points). The drip losses in both sexes were influenced by the feeding method (*p* ≤ 0.01). No drip losses were observed in the muscles of both sexes fed hybrid rye, which may have been associated with the higher pH_24_ value, which was above 6.0.

Muscle loss in cooking was significantly influenced by the sex–feed type interaction (*p ≤* 0.01). Clearly higher loss was observed in the muscles of female geese fed with oats (38.3%) than those fed hybrid rye or a mixture of both grains. Whereas in the ganders’ group this dependence was the opposite, ganders fed oats had a significantly lower loss rate in cooking than the ganders of the other feeding groups.

The results of the basic chemical composition (Table 7) showed a significant effect of feeding on the fat and protein level (*p* ≤ 0.01). Clearly a higher fat content was observed in the muscles of ganders and female geese fed a mixture of both grains (5.10% and 4.83%, respectively) as compared with birds fed hybrid rye only or oats only (3.6% on average). The higher protein content was confirmed in the muscles of both sexes of birds fed hybrid rye than those fed oats (by approximately 0.9 pp) or the grain mixture (by approximately 1.8 pp).

The sensory test results of the cooked breast muscles are presented in Table 8. A statistically significant sex–feed type interaction was observed in the flavour test which indicated that the muscle flavour of geese fed the hybrid rye and oat mixture was less intense (4.05 points) than that of both sexes fed either hybrid rye only or oats only (on average approximately 4.35 points). Feeding had an impact on the remaining organoleptic features, i.e., juiciness, tenderness, and palatability but only in the young female geese. The lowest values for these traits were in the group fed the grain mixture. The sheer force measurements using device did not confirm the differences in the meat tenderness sensory tests results because they ranged between 15.32 and 17.49 N in all of the tested groups. An interesting fact was observed in terms of gender effect on the sensory test results. Feeding the geese hybrid rye or oats showed no differences between the genders in terms of sensory traits but, in the group fed a mixture of these two grains, female geese muscles obtained lower scores than ganders’ muscles in the meat flavour, taste, juiciness, and tenderness tests.

## 4. Discussion

### 4.1. The Effect of Gender and Feeding on the Slaughter Value and Physical Traits of Meat

The results showed that ganders’ weight before slaughter as well as carcass weight was 10–15% higher than in female geese but gender did not have an effect on the slaughter yield, which ranged among the groups from 63% to 65%. No significant differences between the carcass tissues obtained from dissection according to gender and feeding method were found, except for leg muscle weight and skin with fat weight. Similar results were obtained by Kapkowska et al. [7] who reported a higher body weight of the male Zatorska and white Kołudzka varieties fed oats up to 17 weeks of life. The authors did not find any significant differences in slaughter yield between the genders, reaching approximately 64%. Nevertheless, in this study, the birds of both genders fed oats obtained a significantly higher slaughter yield by approximately 2 pp as compared with the group fed hybrid rye.

A higher ganders’ carcass weight was followed by significantly higher weight of carcass elements but there were no differences in their percentage share in the carcass between the two sexes. The meat weight from legs was higher in ganders than in female geese for all groups (*p ≤* 0.01) and in females it was only higher if they were fed oats rather than fed hybrid rye or the mixture of both grains (*p ≤* 0.01). In the group of female geese fed the grain mixture, the abdominal fat content was significantly higher (*p ≤* 0.05) than in ganders’ group. The share of meat from the leg was lower only in those female geese fed the grain mixture (*p ≤* 0.05). No significant differences were found in the percentage share of breast muscles between the sexes or in skin with a fat share. The results obtained by Kapkowska et al. [7] confirmed a higher yield of breast muscles in male carcasses as compared with female carcasses in both of the Zatorska and white Kołudzka geese.

No significant differences between the sexes were reported in terms of the tested physical breast muscle features such as colour lightness and its’ a* and b* parameters, meat marbling and drip loss in all feeding groups. A significantly higher pH_24_ (*p ≤* 0.05) value was observed only in ganders from the group fed oats than in female geese, reaching 5.95 and 5.88, respectively. There were no one-way differences between the sexes in meat cooking losses (significant sex–feed type interaction, *p ≤* 0.01). In the groups fed hybrid rye or the grain mixture, ganders showed higher thermal losses than geese in the group fed oats, which is difficult to explain and justify. The fairly high pH_24_ of goose meat was confirmed by the research of Kapkowska et al. [7] in which the value in both sexes of the Zatorska variety reached 6.09 on average. Additionally, meat colour was pretty dark (L = 38.5), similar to the results obtained in this research (L = approximately 36–37). In addition, the muscles of the geese of the southern varieties presented similar L* values but with a better, lower pH_24_, reaching 5.78 on average [3].

No significant gender effect on the basic chemical composition of breast muscle was reported in any of the feeding groups. The average water content was approximately from 70 to 70.7%, fat content was approximately 3.6–3.8% (except for the group fed the grain mixture, which was between 4.8% and 5.1%) and protein content was approximately 23–25%. In a study by Biesiada-Drzazga [23] on the meat of White Kołudzka geese, the following parameters were observed in birds which were 10 weeks old: protein content of 21% and fat content between 3.1% and 5.1%, depending on the feeding group. The authors of other publications [6,21,24] depending on the breed, origin, and the diet reported on average 19% to 24% protein and 2.3% to 6.3% fat content, in the breast muscle.

Gender did not have any impact on all of the studied sensory traits (flavour, taste, juiciness, and tenderness) of breast muscle in groups fed hybrid rye or oats, but it did have an effect in the case of hybrid rye and oat mixture feeding. The ganders in this feeding group obtained better scores in flavour (*p ≤* 0.01), juiciness (*p ≤* 0.05), taste (*p ≤* 0.01), and tenderness (*p ≤* 0.05) than in females and the difference in scores was approximately 0.3 points. The average results of the sensory tests for the studied features ranged from 3.80 to 4.35 points. Better results in the sensory tests of the southern variety geese meat were reported by Lewko et al. [11] who obtained 4.8 points in cooked breast muscles.

A high score, approximately 4 points, in meat tenderness estimated by the sensory test was confirmed by measurements performed with a device measuring the shear force with low values (from 15.30 to 17.49 N), which did not depend on the gender. In contrast, Kapkowska et al. [7] observed significantly higher shear force values (43.2 to 50.2 N) but, as confirmed in the literature, they were still within the range of good meat tenderness, i.e., approximately 50 N [21].

### 4.2. The Effect of Feeding on Slaughter Traits and Geese Meat Quality

The different feeding groups did not show differences in terms of body weight, but the geese fed hybrid rye obtained approximately a 2 pp lower slaughter yield than those geese fed oats (*p ≤* 0.01 in males and *p ≤* 0.05 in females). The higher slaughter yield in geese fed oats could have been influenced by the higher weight of fat with skin in female geese by approximately 80 g and in ganders by 63 g as compared with the group fed hybrid rye.

Basically, the feeding method did not have any impact on body weight or on the share of carcass elements. The only exception was the weight of geese legs (*p ≤* 0.01) and the percentage share of wings in males (*p ≤* 0.05) from the group fed oats. The highest leg weight of female geese was observed in the oat group, whereas the wing share of males was the highest in the hybrid rye group. The feeding method did not have an effect on the weight and percentage share of breast muscles and share of leg muscles, as well as fat with skin percentage and share and weight of abdominal fat. Other authors have obtained different results. According to a study by Kapkowska et al. [7], the carcasses of Zatorska and White Kołudzka geese had a higher fat content with skin share as compared with the results obtained in this study. Additionally, in a study by Biesiada-Drzazga [2], the abdominal fat share in White Kołudzka geese fed a concentrated feed ranged from 4.2% to 5.8% which indicated a higher fat share than that observed in the geese within this research (2.04% to 2.94% on average). This was also confirmed by Karwowska et al. [25] where the fat share ranged between 6% and 8.5% in the carcasses of White Kołudzka geese fed corn silage and beet pulp, as well as another study by Kokoszyński et al. [26] that applied feeding a corn mixture with 20% addition of oats.

Among the estimated physical features, feeding had an influence on the lower pH_24_ value of breast muscles of female geese fed oats, meat marbling (the highest observed in feeding oats, the lowest in feeding hybrid rye), drip loss (no drip with hybrid rye feeding and approximately 1.3% to 2.0% with oats and mixed grain feeding), as well as the thermal losses of breast muscle samples (the lowest in the oat fattening of ganders and the highest in female geese of this oats group). The changes were not directed one-way (i.e., significant sex–feed type interaction). No effect of the feeding method on colour lightness L* and redness and yellowness parameters was observed but this meat colour should be described as fairly dark (L* = approximately 36), which was also confirmed by Kapkowska et al. [7] and Okruszek et al. [27] who reported average L* = 38.5 for geese breast muscles. However, Lewko et al. [11] observed a lighter colour (L = 44.2) in the breast muscles of southern geese varieties fed oats. Low drip loss was also reported by Kapkowska et al. [7] in 17-week-old geese after feeding oats (0.5%), as well as by Biesek et al. [1] in geese fed a mixture with lupin (0.33% to 0.63%), which are also confirmed in this study. Slightly lower thermal losses in cooking (approximately 30% to 32%), as in this investigation, were observed by Gumułka et al. [8] and Kapkowska et al. [7] in the breast muscles of Zatorska geese after being fed oats.

A basic chemical composition analysis proved the significant effect of feeding on fat and protein content. The highest fat content was observed in the muscles of geese and ganders, which were both fed a grain mixture (4.83% and 5.13%, respectively) as compared with those fed hybrid rye or oats (3.60% on average). These observations were confirmed by the results of visual marbling estimation.

Biesiada-Drzazga [24] reported that, in the breast muscle of White Kołudzka geese fed until 10 weeks of age a concentrated feed with soy and sunflower meal, fat content levels reached 5.1%, 4.3%, and 3.1%, in different experimental groups, and the protein content was 21% on average; the significant effect of feeding on protein content was observed in the studied population. In the muscles of birds fed hybrid rye, a significantly higher protein content, approximately 2 pp, was observed in both genders as compared with birds fed a mixture of two both grains. The muscle protein content in female geese and ganders fed oats was similar (24% on average).

The authors of other publications [6,21,22,25] depending on the geese breed, origin, and diet have reported protein contents on average from 19% to 24% and fat contents from 2.3% to 6.3% in breast muscle. In this study, we confirmed that feeding oats or hybrid rye did not affect the chemical composition of the meat but caused the relatively high protein content and lower fat content.

The results of sensory tests in terms of flavour, juiciness, tenderness, and palatability of cooked meat ranged from 3.8 to 4.35 points. Among these features, there was one statistically significant sex–feed type interaction in the flavour estimation (*p ≤* 0.01). The muscles of female geese had the lowest score (4.05), whereas the goslings in three feeding groups presented, on average, the same values within this parameter (approximately 4.3 points). A statistically significant (*p ≤* 0.01) feeding impact on meat juiciness and the palatability of female geese was observed. Better scores were observed in the meat of female geese fed hybrid rye (over 4 points). Lewko et al. [11] estimated the sensory traits of southern varieties of goose meat depending on the origin, gender, and diet. Cooked breast muscles obtained 4.8 points for the estimated sensory features.

Meat tenderness measurements using devices did not show the effect of feeding on the shear force that fit within 15.3 and 17.49 N. These data proved the good tenderness of the tested meat. Kapkowska et al. [7] reported a higher shear force for the breast muscle of the Zatorska and White Kołudzka varieties, ranging from 43.2 to 50.2 N and Karwoska et al. [9] obtained, for the same musles of the White Kołudzka variety, a mean of 49.8 N.

In summary, it should be stated based on available studies [28,29] the direction for the development of rye genetics should be to create the new cultivars with reduced levels of antinutritional substances. The high level of monosaccharides, as the best source of metabolic energy, could also contribute to an increase in feed intake. The favourable proportions of carbohydrates and lipids in hybrid rye could also determine its nutritional value and possibility have a positive effect on carcass quality. Additionally, the using of hybrid rye in animal feeding could bring economic benefits, because the price of the rye is lower than the other grains [27].

## 5. Conclusions

Our results show the impact of gender on some slaughter value traits and meat quality of young geese slaughtered at 17 weeks old. We found that ganders’ carcass weight and their live weight before slaughter was 10–15% higher than that of female geese. The slaughter yield and the share of cuts and same dissection elements such as breast muscles and skin with fat did not differ between sex. No significant differences were found between genders in colour lightness and a* and b* colour parameters, as well as marbling, drip losses, and basic chemical composition of breast muscle. The sensory estimation results of ganders were better than young female geese only in the case of those ganders fed hybrid rye and a mixture of oats and hybrid rye.

During the last three weeks before slaughter, the type of feeding (hybrid rye, oats, or hybrid rye and oats mixture) did not have a significant effect on geese body weight, but the birds fed hybrid rye had approximately a 2 pp lower slaughter yield than the geese fed oats. The feeding type did not have an influence on the male carcass weight and the share of majority cuts in both sexes, but it had an effect on pH_24_ (it was lower in female geese fed oats), drip losses (none in hybrid rye feeding), marbling (higher in feeding with oats), cooking losses (significant sex–feed type interaction), fat content (higher in mixture feeding), and protein content (higher in hybrid rye feeding). Better sensory estimation results of breast muscles were observed in the young female geese fed hybrid rye or oats.

The obtained results indicate that hybrid rye may be used in the feeding of young geese without causing any negative effects on the final body weight and as a result improving some meat quality traits.

## Figures and Tables

**Table 1:** The experimental plan of the Zatorska geese breed fed different kinds of cereals.

Weeks of Being Reared	Sample Size (n)	Feeding Method
1 to 3	100 nestlings	Feed and water ad libitum, composition of the concentrate and its nutritive value is shown in Table 2
4 to 14	100 birds	Feed and water ad libitum, access in pasture for at least 8 h per day, composition of the concentrate and its nutritive value is shown in Table 2, at the end of 14th week the birds were weighed and divided into 3 feed groups of males and 3 groups of females.
15 to 17	Group A, males *n* = 12 and females *n* = 12	Fed only hybrid rye Brasetto cultivare, birds fed ad libitum with access to straw aviary
	Group B, males *n* = 12 and females *n* = 12	Fed oats, birds fed ad libitum with access to straw aviary
	Group C, males *n* = 12 and females *n* = 12	Concentrate of oats and hybrid rye Brasetto cultivare (1:1, by weight), birds fed ad libitum with access to straw aviary

**Table 2:** Composition of concentrates and chemical composition of feed used in geese feeding (%).

Feeding Component %	Feeding Period	
	0–3 Weeks	4–14 Weeks
Feed phosphate	1.0	1.0
Limestone	1.8	1.2
Maize	40.0	35.0
Premix	1.2	1.1
Wheat	27.5	24.2
Wheat bran	-	10.0
Soy bean meal	28.5	23.5
Sunflower extracted meal	-	4.0
Crude protein	19.5	19.2
Crude fibre	2.8	3.8
Vegetable oils and crude fat	2.5	2.6
Crude ash	5.4	5.1
Lysine	0.97	0.91
Methionine	0.48	0.40
Calcium	0.94	0.73
Sodium	0.17	0.17
Available phosphorus	0.36	0.44
Metabolic energy, MJ/kg feed	11.50	10.20

**Table 3:** Mean of the slaughter traits of geese fattened by feeding hybrid rye or oats.

Specification	Fattening Groups			SEM	*p*-Value for	
	Rye A	Oats B	Rye/Oats C		Fattening	Interaction
Pre-slaughter weight (g)						
Males	5341.25	5444.58	5460.42	70.25	0.764	0.597
Females	4617.92	4905.00	4715.00	57.14	0.105	
SEM	109.38	102.56	100.92			
*p*-Value for sex	0.000	0.006	0.000			
Carcass weight (g)						
Males	3368.80	3580.00	3500.20	54.88	0.292	0.800
Females	2931.50 b	3226.30 a	3036.80	47.35	0.031	
SEM	74.06	82.43	63.41			
*p*-Value for sex	0.001	0.028	0.000			
Dressing percentage (%)						
Males	63.02 B	65.64 A	64.12	0.35	0.006	0.949
Females	63.43 b	65.68 a	64.60	0.39	0.039	
SEM	0.34	0.48	0.34			
*p*-Value for sex	0.561	0.963	0.505			

N = 12 in every experimental group males and females; the letters A, B, C mean the fattening groups, i.e., (A) birds fed hybrid rye, (B) birds fed oats, (C) birds fed a mixture of hybrid rye/oats (1:1); SEM, standard error of the means; means marked with a letter (A, B, C or a, b, c) represents the fattening group with which it differs statistically, means marked by lowercase (a, b, c) are significant at *p* ≤ 0.05 and means marked by uppercase (A, B, C) are significant at *p* ≤ 0.01, the lack of a letter means that the mean does not statistically differ with any other fattening group.

**Table 4:** Weight and the proportion of parts in geese carcasses.

Specification	Fattening Groups			SEM	*p*-Value for	
	Rye A	Oats B	Rye/Oats C		Fattening	Interaction
**Neck with skin (g)**						
Males	209.90	212.9	211.10	4.52	0.963	0.992
Females	172.80	177.1	175.70	3.22	0.867	
SEM	6.07	6.05	6.26			
*p*-Value for sex	0.001	0.001	0.002			
**Neck with skin (%)**						
Males	6.23	5.95	6.02	0.09	0.417	0.758
Females	5.91	5.49	5.78	0.08	0.108	
SEM	0.12	0.09	0.11			
*p*-Value for sex	0.180	0.001	0.284			
**Wing (g)**						
Males	537.80	538.80	536.10	8.68	0.992	0.677
Females	451.60	475.90	457.20	6.53	0.289	
SEM	12.97	12.18	11.72			
*p*-Value for sex	0.000	0.007	0.000			
**Wing (%)**						
Males	15.98 b	15.09 a	15.30	0.15	0.040	0.718
Females	15.40	14.79	15.06	0.11	0.089	
SEM	0.13	0.19	0.15			
*p*-Value for sex	0.022	0.435	0.436			
**Legs (g)**						
Males	687.40	730.80	682.40	12.97	0.253	0.808
Females	578.70 b	640.50 ac	567.3 b	10.89	0.009	
SEM	18.28	16.67	17.49			
*p*-Value for sex	0.001	0.004	0.000			
**Legs (%)**						
Males	20.39	20.46	19.52	0.25	0.236	0.933
Females	19.79	19.94	18.67	0.28	0.135	
SEM	0.31	0.32	0.33			
*p*-Value for sex	0.350	0.431	0.202			
**Body frame (g)**						
Males	839.30	885.20	861.80	20.22	0.665	0.921
Females	724.90	783.30	734.30	15.90	0.281	
SEM	21.48	28.31	24.79			
*p*-Value for sex	0.005	0.071	0.007			
**Body frame (%)**						
Males	24.92	24.79	24.59	0.44	0.957	0.970
Females	24.74	24.24	24.24	0.40	0.847	
SEM	0.37	056	0.60			
*p*-Value for sex	0.812	0.635	0.777			

N = 12 in every experimental group males and females; the letters A, B, C mean the fattening groups, i.e., (A) birds fed hybrid rye, (B) birds fed oats, (C) birds fed a mixture of hybrid rye/oats (1:1); SEM, standard error of the means; means marked with a letter (A, B, C or a, b, c) represents the fattening group with which it differs statistically, means marked by lowercase (a, b, c) are significant at *p* ≤ 0.05 and means marked by uppercase (A,B,C) are significant at *p* ≤ 0.01, the lack of a letter means that the mean does not statistically differ with any other fattening group. Additionally, if the mean is signed by two letters “ac”, that means significant difference as compared with means of both signed fattening groups a and c.

**Table 5:** Mean of weight and percentage of chosen muscles and fat obtained from the dissection of geese carcasses.

Specification	Fattening Groups			SEM	*p*-Value for	
	Rye A	Oats B	Rye/Oats C			
					Fattening	Interaction
**Breast muscle (g)**						
Males	520.20	579.20	571.4	14.28	0.184	0.631
Females	489.30	537.90	495.3	14.35	0.330	
SEM	15.21	20.67	17.11			
*p*-Value for sex	0.322	0.321	0.023			
**Breast muscle %**						
Males	15.40	16.12	16.36	0.26	0.295	0.497
Females	16.66	16.65	16.29	0.36	0.903	
SEM	0.33	0.37	0.46			
*p*-Value for sex	0.058	0.488	0.949			
**Legs muscles (g)**						
Males	431.60	461.60	442.10	9.66	0.450	0.700
Females	357.50 b	394.60 c	350.70 b	7.75	0.040	
SEM	13.43	12.66	13.21			
*p*-Value for sex	0.003	0.005	0.000			
**Legs muscles %**						
Males	12.79	12.91	12.65	0.21	0.885	0.721
Females	12.24	12.30	11.54	0.23	0.332	
SEM	0.27	0.29	0.29			
*p*-Value for sex	0.313	0.300	0.042			
**Skin with subcutaneous fat (g)**						
Males	473.80	537.4	557.1	16.46	0.094	0.877
Females	435.80 b,c	515.7 a	509.4 a	14.56	0.040	
SEM	16.47	20.97	16.39			
*p*-Value for sex	0.258	0.615	0.150			
**Skin with subcutaneous fat %**						
Males	14.08	14.98	15.92	0.26	0.295	0.497
Females	14.84	15.93	16.73	0.36	0.903	
SEM	0.43	0.47	0.44			
*p*-Value for sex	0.393	0.321	0.374			
**Abdominal fat (g)**						
Males	90.80	85.60	71.80	5.62	0.371	0.119
Females	70.10	83.40	89.30	4.93	0.274	
SEM	7.51	5.32	6.51			
*p*-Value for sex	0.174	0.844	0.185			
**Abdominal fat (%)**						
Males	2.70	2.34	2.04	0.16	0.249	0.082
Females	2.37	2.57	2.94	0.15	0.319	
SEM	0.22	0.13	0.21			
*p*-Value for sex	0.472	0.408	0.034			

N = 12 in every experimental group males and females; the letters A, B, C mean the fattening groups, i.e., (A) birds fed hybrid rye, (B) birds fed oats, (C) birds fed a mixture of hybrid rye/oats (1:1); SEM, standard error of the means; means marked with a letter (A, B, C or a, b, c) represents the fattening group with which it differs statistically, means marked by lowercase (a, b, c) are significant at *p* ≤ 0.05 and means marked by uppercase (A,B,C) are significant at *p* ≤ 0.01, the lack of a letter means that the mean does not statistically differ with any other fattening group. Additionally, if the mean is signed by two letters e.g., “ac”, that means significant difference as compared with means of both signed fattening groups a and c.

**Table 6:** Selected physical parameters of breast muscles.

Specification	Fattening Groups			SEM	*p*-Value	
	Rye A	Oats B	Rye/Oats C		Fattening	Interaction
**pH_24_**						
Males	6.04	5.95	6.02	0.02	0.071	0.632
Females	6.03 B	5.88 A	5.96	0.021	0.010	
SEM	0.03	0.01	0.02			
*p*-Value for sex	0.858	0.019	0.121			
Colour L*						
Males	36.52	36.49	35.60	0.32	0.424	0.440
Females	35.67	36.28	36.48	0.44	0.748	
SEM	0.60	0.64	0.39			
*p*-Value for sex	0.369	0.784	0.453			
a* redness						
Males	19.19	19.29	20.15	0.27	0.299	0.671
Females	18.96	18.39	19.10	0.29	0.570	
SEM	0.32	0.34	0.38			
*p*-Value for sex	0.725	0.188	0.170			
b* yellowness						
Males	−1.41	−0.95	−1.37	0.20	0.603	0.854
Females	−1.76	−1.69	−1.21	0.33	0.768	
SEM	0.33	0.29	0.39			
*p*-Value for sex	0.605	0.203	0.835			
**Colour, points**						
Males	3.38	3.26	3.30	0.06	0.669	0.413
Females	3.28 c	3.12	2.97 a	0.05	0.045	
SEM	0.06	0.07	0.07			
*p*-Value for sex	0.439	0.332	0.012			
**Marbling, points**						
Males	1.32 B	1.59 Ac	1.43 b	0.03	0.001	0.543
Females	1.32 Bc	1.60 A	1.53 a	0.04	0.005	
SEM	0.03	0.04	0.04			
*p*-Value for sex	0.990	0.896	0.185			
**Drip loss %**						
Males	0.00 BC	1.34 A	1.55 A	0.19	0.001	0.495
Females	0.00 BC	1.92 A	1.86 A	0.19	0.000	
SEM	0.00	0.26	0.16			
*p*-Value for sex		0.264	0.341			
**Cooking loss %**						
Males	37.32 B	32.07 Ac	35.89 b	0.70		0.000
Females	33.73 B	38.30 AC	33.04 B	0.66		
SEM	0.89	1.01	0.53			
*p*-Value for sex	0.040	0.001	0.004			

N = 12 in every experimental group males and females; the letters A, B, C mean the fattening groups, i.e., (A) birds fed hybrid rye, (B) birds fed oats, (C) birds fed a mixture of hybrid rye/oats (1:1); SEM, standard error of the means; means marked with a letter (A, B, C or a, b, c) represents the fattening group with which it differs statistically, means marked by lowercase (a, b, c) are significant at *p* ≤ 0.05 and means marked by uppercase (A,B,C) are significant at *p* ≤ 0.01, the lack of a letter means that the mean does not statistically differ with any other fattening group. Additionally, if the mean is signed by two letters e.g., “ac”, that means significant difference as compared with means of both signed fattening groups a and c.

**Table 7:** Basic chemical composition of the breast muscles.

Traits	Fettening Groups			SEM	*p*-Value for	
	Rye A	Oats B	Rye/Oats C		Fattening	Interaction
**Water content (%)**						
Males	69.75	70.72	70.34	0.22	0.193	0.976
Females	69.94	70.96	70.69	0.22	0.136	
SEM	0.304	0.242	0.213			
*p*-Value for sex	0.764	0.632	0.429			
**Fat content(%)**						
Males	3.68 C	3.60 C	5.10 AB	0.160	0.000	0.710
Females	3.76 C	3.57 C	4.83 AB	0.157	0.001	
SEM	0.13	0.13	0.17			
*p*-Value for sex	0.764	0.919	0.466			
**Total protein content (%)**						
Males	25.01 C	24.14	23.07 A	0.244	0.003	0.981
Females	24.77 C	23.92	22.96 A	0.250	0.009	
SEM	0.340	0.250	0.160			
*p*-Value for sex	0.727	0.665	0.755			

N = 12 in every experimental group males and females; the letters A, B, C mean the fattening groups, i.e., (A) birds fed hybrid rye, (B) birds fed oats, (C) birds fed a mixture of hybrid rye/oats (1:1); SEM, standard error of the means; means marked with a letter (A, B, C or a, b, c) represents the fattening group with which it differs statistically, means marked by lowercase (a, b, c) are significant at *p* ≤ 0.05 and means marked by uppercase (A,B,C) are significant at *p* ≤ 0.01, the lack of a letter means that the mean does not statistically differ with any other fattening group. Additionally, if the mean is signed by two letters e.g., “ac”, that means significant difference as compared with means of both signed fattening groups a and c.

**Table 8:** Sensory evaluation results (points) and shear force measurements of cooked breast.

Traits	Fattening Groups			SEM	*p*-Value for	
	Rye A	Oats B	Rye/Oats C		Fattening	Interaction
**Flavour (points)**						
Males	4.35	4.31	4.35	0.03		0.000
Females	4.46 C	4.38 C	4.05 AB	0.04		
SEM	0.03	0.04	0.04			
*p*-Value for sex	0.130	0.421	0.000			
**Juiciness (points)**						
Males	3.75	3.88	3.76	0.06	0.648	0.064
Females	3.88 bC	3.61 a	3.54 A	0.04	0.004	
SEM	0.06	0.08	0.05			
*p*-Value for sex	0.325	0.092	0.037			
**Tenderness (points)**						
Males	3.89	3.95	3.95	0.04	0.813	0.069
Females	4.03 c	3.93	3.70 a	0.05	0.036	
SEM	0.07	0.06	0.06			
*p*-Value for sex	0.287	0.860	0.025			
**Palatability (points)**						
Males	4.10	4.05	4.02	0.04	0.687	0.092
Females	4.09 C	4.05 C	3.76 AB	0.04	0.001	
SEM	0.05	0.05	0.04			
*p*-Value for sex	0.925	1.00	0.002			
**Shear force (N)**						
Males	17.09	17.49	16.62	0.44	0.738	0.754
Females	15.32	16.01	15.84	0.40	0.755	
SEM	0.55	0.64	0.36			
*p*-Value for sex	0.111	0.251	0.358			

N = 12 in every experimental group males and females; the letters A, B, C mean the fattening groups, i.e., (A) birds fed hybrid rye, (B) birds fed oats, (C) birds fed a mixture of hybrid rye/oats (1:1); SEM, standard error of the means; means marked with a letter (A, B, C or a, b, c) represents the fattening group with which it differs statistically, means marked by lowercase (a, b, c) are significant at *p* ≤ 0.05 and means marked by uppercase (A,B,C) are significant at *p* ≤ 0.01, the lack of a letter means that the mean does not statistically differ with any other fattening group. Additionally, if the mean is signed by two letters e.g., “ac”, that means significant difference as compared with means of both signed fattening groups a and c.

## Data Availability

Not applicable.
